# Circulating innate lymphoid cells and IL-18 as potential immune biomarkers in thymic tumors

**DOI:** 10.3389/fimmu.2025.1705648

**Published:** 2025-11-24

**Authors:** Daniela Claudia Maresca, Maria Rosaria Saponaro, Evelina La Civita, Marianna Tortora, Benedetta Romano, Erica Pietroluongo, Fabio Somma, Mario Giuliano, Alberto Servetto, Giovannella Palmieri, Angela Ianaro, Daniela Terracciano, Giuseppe Ercolano

**Affiliations:** 1 Department of Pharmacy, School of Medicine, University of Naples Federico II, Naples, Italy; 2 Department of Clinical Medicine and Surgery, University Federico II, Naples, Italy; 3 Department of Translational Medical Sciences, University of Naples Federico II, Naples, Italy; 4 Rare Tumors Coordinating Center of Campania Region (CRCTR), University Federico II, Naples, Italy

**Keywords:** thymic epithelial tumors, innate lymphoid cells, thymoma, thymic carcinoma, ILCs

## Abstract

**Introduction:**

Thymic epithelial tumors (TETs) are rare malignancies frequently associated with autoimmunity. However, circulating immune biomarkers for patient stratification and disease monitoring remain undefined. Innate lymphoid cells (ILCs) are emerging regulators of tumor immunity, but their role in TETs has not yet been characterized.

**Methods:**

Peripheral blood samples from 32 patients with histologically confirmed TETs and 20 healthy donors were analyzed by multiparametric flow cytometry to quantify circulating ILC subsets. Serum cytokine concentrations were measured using multiplex immunoassays. Patients were stratified according to histology, disease activity, and presence of autoimmune manifestations.

**Results:**

TETs displayed a significant expansion of circulating ILCs, mainly driven by an enrichment of ILC1, which was more pronounced in patients with active disease and in those with thymic carcinoma. Serum IL-18 levels were elevated—particularly in thymic carcinoma—and correlated with higher concentrations of type 2 cytokines (IL-4, IL-5, IL-9, IL-13). No concomitant increase in canonical ILC1 effector cytokines, including IFN-γ, was observed, indicating a functional dissociation between ILC1 expansion and their expected cytokine profile.

**Discussion:**

These findings delineate a distinct systemic immune signature in TETs, characterized by IL-18 upregulation and altered ILC1 dynamics, with potential implications for immune regulation and autoimmunity. Circulating ILC profiling combined with IL-18 measurement may represent a promising approach for patient stratification, disease monitoring, and the development of novel immunomodulatory strategies in TETs.

## Introduction

Thymic epithelial tumors (TETs) represent a rare and heterogeneous group of neoplasms arising from the epithelial cells of the thymus, a primary lymphoid organ essential for the maturation of T lymphocyte precursors. Although they account for only 0.2% to 1.5% of all cancers, TETs represent a significant proportion of tumors in the anterior mediastinum, with an estimated incidence of approximately 0,15 cases per 100,000 individuals annually (1, 2). TETs encompass thymomas (T), thymic carcinoma (TC), and neuroendocrine tumors of the thymus. Given the thymus’s central role in immune system development and regulation, TETs are frequently associated with autoimmune disorders such as myasthenia Gravis (MG) and Good’s syndrome (GS) (3). Understanding the biological and immunological features of TETs is crucial to improving clinical management and exploring novel therapeutic strategies for these rare tumors. Innate lymphoid cells (ILCs) are tissue-resident lymphocytes considered the innate counterparts of CD4^+^ T helper cells. They comprise three main subsets (ILC1, ILC2, and ILC3) with distinct transcription factors and cytokine profiles, and exert context-dependent roles in cancer immunity influenced by the tumor microenvironment (4, 5). Depending on the tumor context, ILC subsets can either promote or suppress tumor growth, reflecting their remarkable functional plasticity (6–12). Their plasticity and responsiveness to tumor-derived signals suggest potential value as biomarkers for immune stratification and as targets for immunotherapy. However, their role in TETs remains unexplored. Here, we aimed to characterize the peripheral ILC landscape and related cytokine signatures in patients with TETs, compared to healthy donors, in relation to tumor subtype, disease status, and autoimmunity. Specifically, we examined their phenotypic distribution, quantified the levels of key activating cytokines, and assessed the functional profile of their cytokine production. By characterizing the ILC landscape in TETs, this study aims to shed light on their potential involvement in shaping the tumor immune microenvironment and to identify novel immune targets. Ultimately, these findings may provide new insights into the intricate relationship between innate immunity and tumor development, laying the groundwork for future immune-based therapeutic strategies in the management of TETs.

## Materials and methods

### Study design and participants

This prospective study enrolled consecutive patients with thymic epithelial tumors who were referred to the Regional Coordinating Center for Rare Tumors of the Campania Region, located at the University Hospital Federico II in Naples, Italy, between May 2022 and March 2023. Inclusion criteria were the following: age 18 years or older, histological diagnosis of a thymic epithelial tumor (Thymoma, T; Thymic Carcinoma, TC), known disease status, defined as either evidence of disease (ED) or no residual tumor lesion(s) (NED), known status of autoimmune disorders (presence, AD; absence, NO AD). Diagnosis of autoimmune disorders was defined as per national guidelines (13, 14) using following criteria:

1. Diagnosis of GS was defined by hypogammaglobulinemia, low or absent B cells, abnormal CD4/CD8 T-cell ratio, CD4 T-cell lymphopenia, and impaired T-cell mitogenic responses, associated with increased susceptibility to infections owing to encapsulated bacteria, fungi, or viruses.
2. Diagnosis of MG was defined by the presence of positive antibodies directed against postsynaptic antigens, muscle cholinergic receptor, muscle tyrosine kinase, or LRP4, accompanied or not by clinical signs of ptosis, diplopia, or muscle weakness.
3. Diagnosis of antibody-mediated encephalitis was defined by the presence in plasma and/or cerebrospinal fluid of positive antibodies directed against the α-amino-3-hydroxy-5-methyl-4-isoxazolepropionic acid receptor (AMPAR), Leucin-rich-glioma-inactivated 1 (LGI1), Contacting-associated protein-like2 (Caspr2), gamma-aminobutyric acid type A receptor (GABAR) and variety of alteration in brain magnetic resonance imaging (MRI) associated with neuropsychiatric symptoms as behavioral abnormalities, psychosis, epileptic seizures, dysautonomia and reduced level of consciousness. The autoimmune conditions were observed only in patients with thymoma. Autoimmune syndrome diagnosis and disease status were confirmed for each patient prior to enrolling. For serum collection, whole blood was centrifuged at 10,000g for 10 min, and the upper phase was collected and immediately frozen. PBMCs were isolated by density gradient centrifugation using Ficoll-Paque (1800 RPM for 20 min at room temperature) and immediately cryopreserved in 90% fetal bovine serum (FBS) and 10% dimethylsulfoxide (DMSO).

### Flow cytometry analysis

Human ILCs were identified as lineage (Lin) negative and CD127 positive cells. Lineage markers, all FITC-conjugated, include: anti-human CD4 (RPA-T4, SONY, 1:400), anti-human CD8 (REA734, Milteny, 1:800), anti-human CD14 (REA599, Milteny, 1:800), anti-human CD15 (HI98, SONY, 1:1.600), anti-human CD20 (REA780, Milteny, 1:400), anti-human CD33 (HIM3-4, SONY, 1:400), anti-human CD34 (561, SONY, 1:800), anti-human CD203C (NP4D6, SONY, 1:100), anti-human FCεRIα (AER-37 (CRA-1), BioLegend, 1:800). The Zombie Green viability dye (Biolegend), included in the FITC channel together with lineage markers, was used to exclude dead cells. Additional lineage markers are: PERCP 5.5 conjugated including anti-human CD3 (OK73, BioLegend, 1:800) and anti-human CD19 (HIB19, BioLegend, 1:800), PE-CY7 anti-human CD16 (3G8, BioLegend, 1:100) and CD56 (5.1H11, BioLegend, 1:100), while anti-human CD127 antibody was APC-CY7 conjugated (A019D5, SONY, 1:100). Additional markers used to identify ILC subsets were: anti-human CD117 (cKit) in APC (S18022G, BioLegend, 1:100) and anti-human CRTH2 in PE (BM16, SONY, 2:50); specifically, ILC1s were identified as a double-negative population, ILC2s as a CRTH2^+^CD117^+/-^ and ILCPs as a CRTH2^-^CD117^+^ population.

Human CD4 T cell subsets were identified using the following antibodies: APC-CY7 anti‐human CD45RO (UCHL1, Biolegend, 1:50), PC7 anti‐human CXCR3 (1C6, Biolegend, 2:50), APC anti‐human CD196 (CCR6) (G034E3, Biolegend, 2:50). Th subsets were identified as follow: Th1 as CXCR3^+^CRTH2^−^CCR6^−^, Th2 as CRTH2^+^, Th17 as CXCR3^−^CRTH2^−^CCR6^+^. Cells were resuspended in FACS buffer and analyzed using a BriCyte E6 flow cytometer (Mindray Medical Italy S.r.l., Milan, Italy). The data were analyzed using FlowJo software (TreeStar V.10; Carrboro, NC, USA).

### Serum cytokine quantification

Serum cytokine levels were measured using BioLegend’s LEGENDplex™ bead-based immunoassays. Specifically, for TSLP, GM-CSF, IL-23, IL-15, IL-18, IL-11 and IL-33 the Human Cytokine Panel 2 (13-Plex) was used, while for IL-5, IL-13, IL-9, IL-4, and Th cytokine panels (12-plex). Analyses were performed according to the manufacturer’s instructions.

### Correlation analysis

Correlations between circulating ILC subset frequencies, plasma cytokine levels, and clinical parameters were assessed using Spearman’s rank correlation. Clinical categorical variables were numerically encoded as follows: sex (0 = female, 1 = male), autoimmunity (0 = absent, 1 = present), disease status (0 = NED, 1 = ED), and histology (0 = thymoma, 1 = thymic carcinoma). Correlation coefficients were visualized as a color-coded heatmap (blue = positive; red = negative).

### Statistical analysis

GraphPad Prism 9 software was used for statistical analyses. Paired or unpaired t-test were used when comparing two groups. ANOVAs or the non-parametric Kruskal-Wallis test were used for comparison of multiple groups. Data in graphs represent the mean ± SEM, with a p value <0.05 (two-tailed) being significant and labeled with *. P values <0.01, <0.001 or <0.0001 are indicated as **, *** and ****, respectively.

## Results

### ILCs frequency is altered in patients with TETs compared to healthy donors

Peripheral blood samples were collected from 32 patients with histologically confirmed thymic epithelial tumors (TETs), including 20 with thymoma (62.5%), 10 with thymic carcinoma (31.3%), and 2 with both subtypes (6.2%). Fourteen patients (43.6%) presented autoimmune comorbidities. At the time of sampling, 17 patients (53.2%) showed no evidence of disease (NED), while 15 (46.8%) had evidence of disease (ED). Detailed clinical characteristics are provided in **Table 1**. At time of enrolling, most patients in the thymic carcinoma subgroup were undergoing systemic treatment, while a few blood samples were collected before starting therapy. specifically: 1 patient was NED under post-surgical surveillance. Among ED cases, 2 were sampled pre-chemotherapy, 1 was on etoposide plus a somatostatin analogue, 1 on ramucirumab, 2 on carboplatin–paclitaxel, 2 on cisplatin–doxorubicin cyclophosphamide, and 1 was under radiological surveillance for stable disease. In the thymoma subgroup, however, most patients were NED under surveillance; among ED cases, 3 were on etoposide, 1 was on somatostatin analogue and 1 was on cisplatin–doxorubicin–cyclophosphamide. In the thymoma subgroup with autoimmune disorders, 11/14 patients were receiving corticosteroids.

**Table 1:** Patient characteristics.

Characteristics	Number of patients (%)
Age, median (interval)	55 (31–72)
Sex:	
Female	14 (43.6%)
Male	18 (56.4%)
Histologic type	
Thymoma	20 (62.5%)
A	2 (6.2%)
AB	3 (9.5%)
B (B1, B2, B3)	15 (46.8%)
Thymic carcinoma	10 (31.3%)
Thymoma B3+Thymic carcinoma	2 (6.2%)
Autoimmune disorders*	
Yes	14 (43.6%)
GS	5 (15.6%)
MG	7 (21.8%)
Encephalitis	1 (3.1%)
GS+MG	1 (3.1%)
No	18 (56.4%)
Disease	
Evidence of disease	15 (46.8%)
No Evidence of disease	17 (53.2%)

*Autoimmune disorders were present exclusively in patients with thymoma.

**Figure 1:**
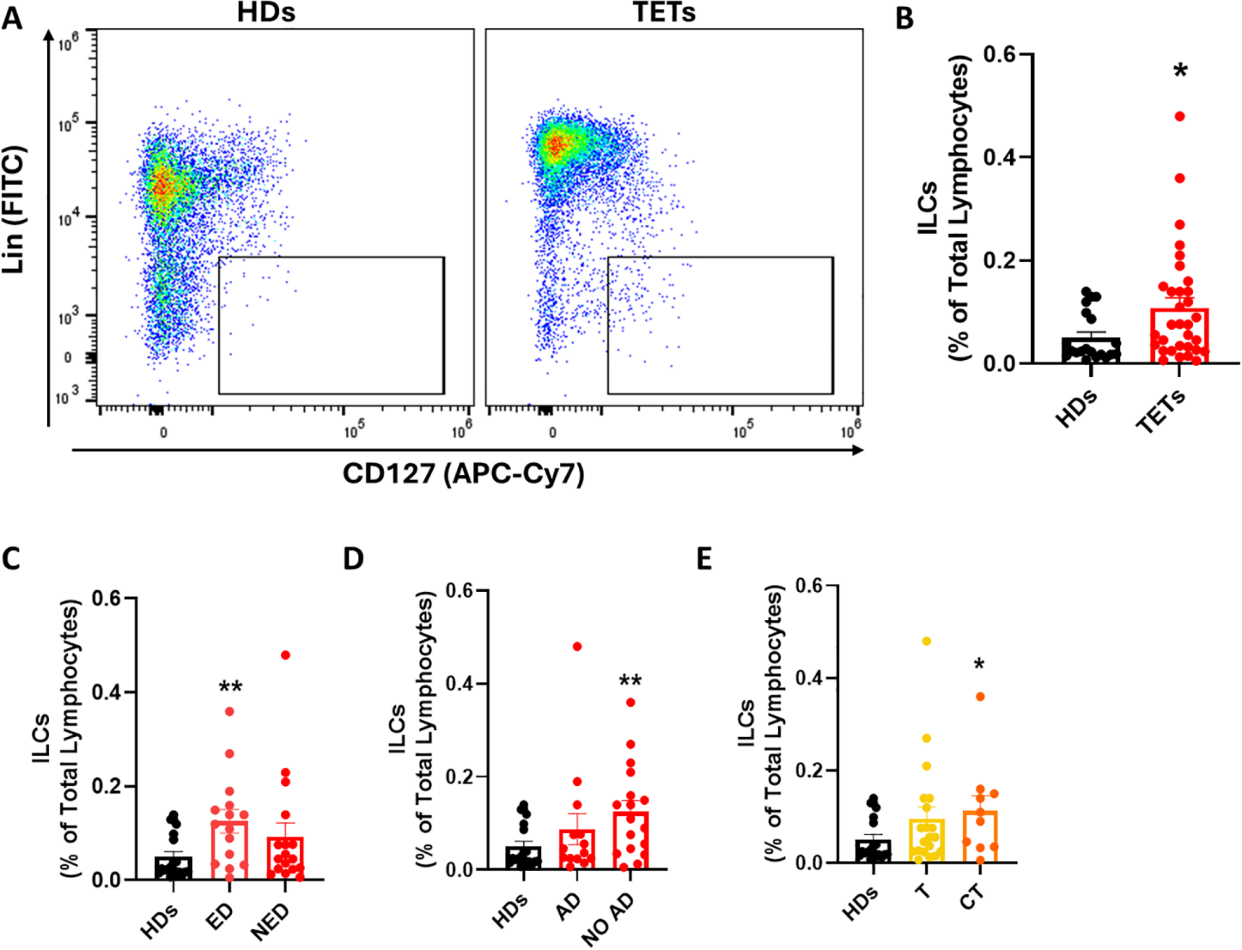
ILCs frequency is altered in patients with TETs compared to healthy donors. **(A)** Representative examples of flow cytometry analysis of total ILCs in PBMCs of HDs (n=20) and TETs patients (n=32). **(B)** Frequency of total ILCs in HD and TETs patients. **(C)** Frequency of total ILCs in patients with evidence of disease (ED; n=15) and without evidence of disease (NED; n=17). **(D)** Frequency of total ILCs in patients with autoimmune disorders (AD; n=14) and without autoimmune disorders (NO AD; n=17). **(E)** Frequency of total ILCs in TETs patients divided in thymoma (T; n=20) and thymic carcinoma (CT; n=10). Data are shown as mean ± SEM (*p < 0.05; **p < 0.01 vs HDs) and were analyzed by Wilcoxon and/or one-way ANOVA tests.

First, we quantified the frequency of total ILCs, defined as lineage^-^ CD127^+^ lymphocytes, within peripheral blood mononuclear cells (PBMCs) using multiparametric flow cytometry (representative gating strategy and FMO controls are shown in **Supplementary Figures 1A, B**). Compared to healthy donors (HDs, *n* = 20), TET patients (*n* = 32) exhibited a significant increase in circulating ILC frequency (**Figures 1A, B**). Subsequently, we stratified patients based on disease status and found that patients with evidence of disease (ED, *n* = 15) had a markedly elevated ILC frequency compared to HDs (**Figure 1C**). Similarly, when stratified by autoimmune status, patients without autoimmune disorders (NO AD, *n* = 18) displayed higher ILC frequencies compared to HDs (**Figure 1D**). Systemic antitumor treatment could influence immune parameters; however, due to the limited sample size, formal adjusted analyses were not feasible. Lastly, when categorized by tumor histology, a significant increase in the frequency of total ILCs was observed in thymic carcinoma (CT, *n* = 10) patients compared to HDs while thymoma patients (T, *n* = 20) exhibited a moderate elevation (**Figure 1E**). These findings point to a systemic dysregulation of ILCs in TETs, potentially influenced by tumor type and disease activity. This systemic expansion may serve as an accessible peripheral biomarker for identifying patients with evidence of disease or histologically aggressive tumors.

### ILC subsets are dysregulated in TET patients

**Figure 2:**
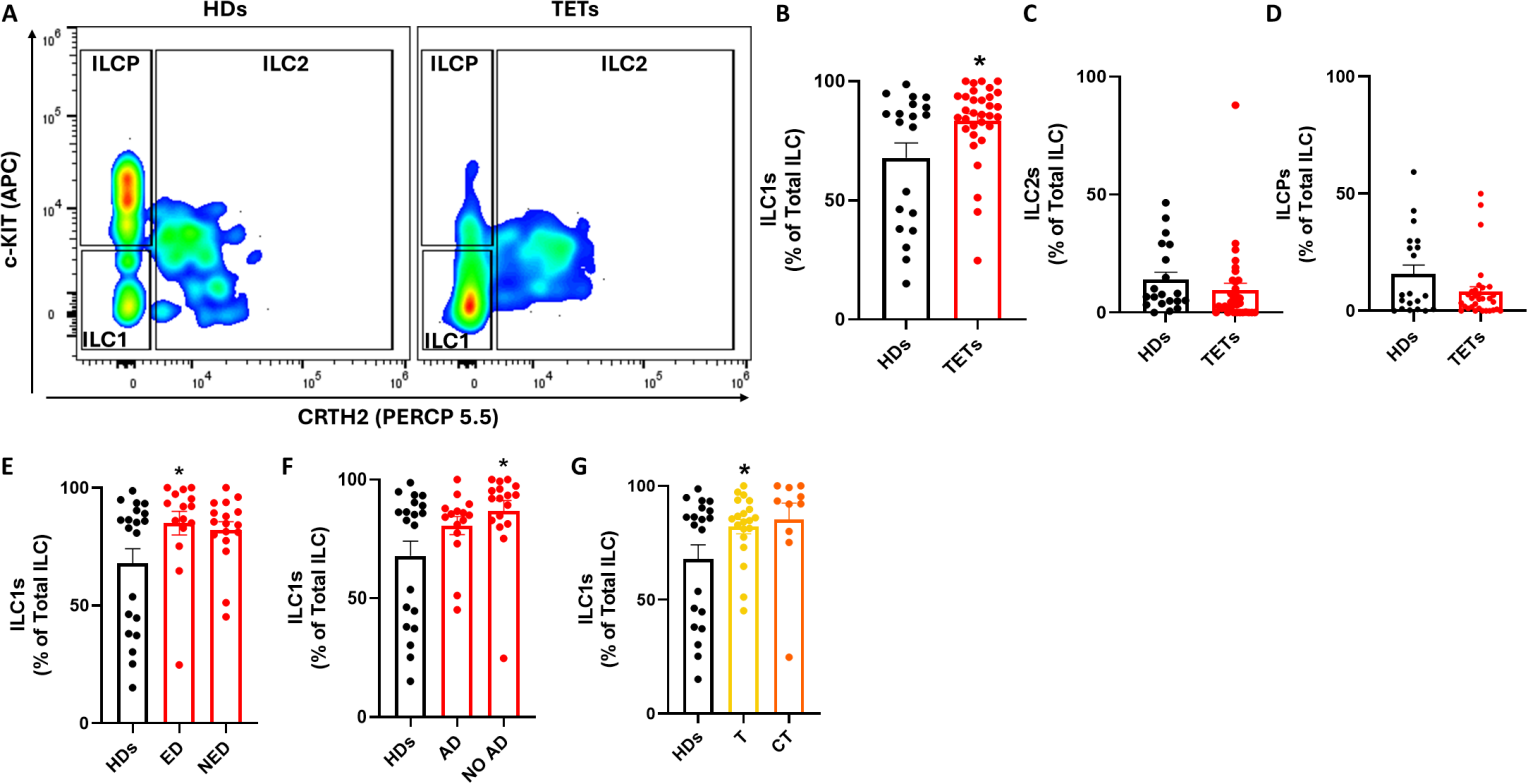
ILCs subsets are dysregulated in TETs patients. **(A)** Representative examples of flow cytometry analysis of ILC subsets in PBMCs of HD (n=20) and TETs patients (n=32). **(B)** Frequency of ILC1s in HD and TETs patients. **(C)** Frequency of ILC2s in HD and TETs patients. **(D)** Frequency of ILCPs in HD and TETs patients. **(E)** Frequency of ILC1s in HDs and in patients with evidence of disease (ED; n=15) and without evidence of disease (NED; n=17). **(F)** Frequency of ILC1s in in HDs and patients with autoimmune disorders (AD; n=14) and without autoimmune disorders (NO AD; n=17). **(G)** Frequency of ILC1s in TETs patients divided in thymoma (T; n=20) and thymic carcinoma (CT; n=10). Data are shown as mean ± SEM (*p <0.05) and were analyzed by Wilcoxon and/or one-way ANOVA tests.

To gain deeper insights into ILC dysregulation in TETs, we next analyzed the distribution of specific ILC subsets, distinguishing ILC1s, ILC2s, and ILCPs based on CRTH2 and c-Kit expression (**Figure 2A**). A significant increase in the frequency of ILC1s was observed in TET patients compared to healthy donors (**Figure 2B**), whereas no significant differences were found for ILC2s or ILCPs (**Figures 2C, D**). Given this selective expansion of ILC1s, we further stratified patients according to disease activity and autoimmune status. ILC1 frequencies were significantly higher in patients with evidence of disease (ED) and in those without autoimmune disorders (NO AD) compared to healthy donors (**Figures 2E, F**). In contrast, ILCP frequencies remained unchanged across these subgroups, whereas ILC2 significantly decreased in NO AD patients (**Supplementary Figure 2**). When stratified by tumor histology, an increase in ILC1s was detected in both thymoma and thymic carcinoma patients; however, statistical significance was reached only in the thymoma group (**Figure 2G**). These findings suggest that among circulating ILC subsets, ILC1s are preferentially expanded in TET patient, particularly in those with evidence of disease (ED), absence of autoimmunity, and thymoma histology. The selective increase in ILC1s, particularly in ED patients, suggests their potential as a dynamic marker for monitoring tumor activity. To assess whether the alterations observed in circulating ILC subsets were mirrored by changes in their adaptive counterparts, we analyzed CD4^+^ Th1, Th2, and Th17 cells in peripheral blood from HD and TET patients (**Supplementary Figure 3**). Th1 frequencies were increased in TET compared with HD, consistent with the expansion of ILC1 and with a polarization toward type-1 inflammation. In contrast, Th17 cells were reduced, whereas Th2 frequencies remained unchanged. Similar to ILC1, Th1 cells displayed the same trend across clinical subgroups, including sex, autoimmunity, disease status, and histology. These results further support that TET are characterized by a systemic bias toward type-1 immunity involving both innate and adaptive compartments.

### IL-18 is selectively increased in TET patients

**Figure 3:**
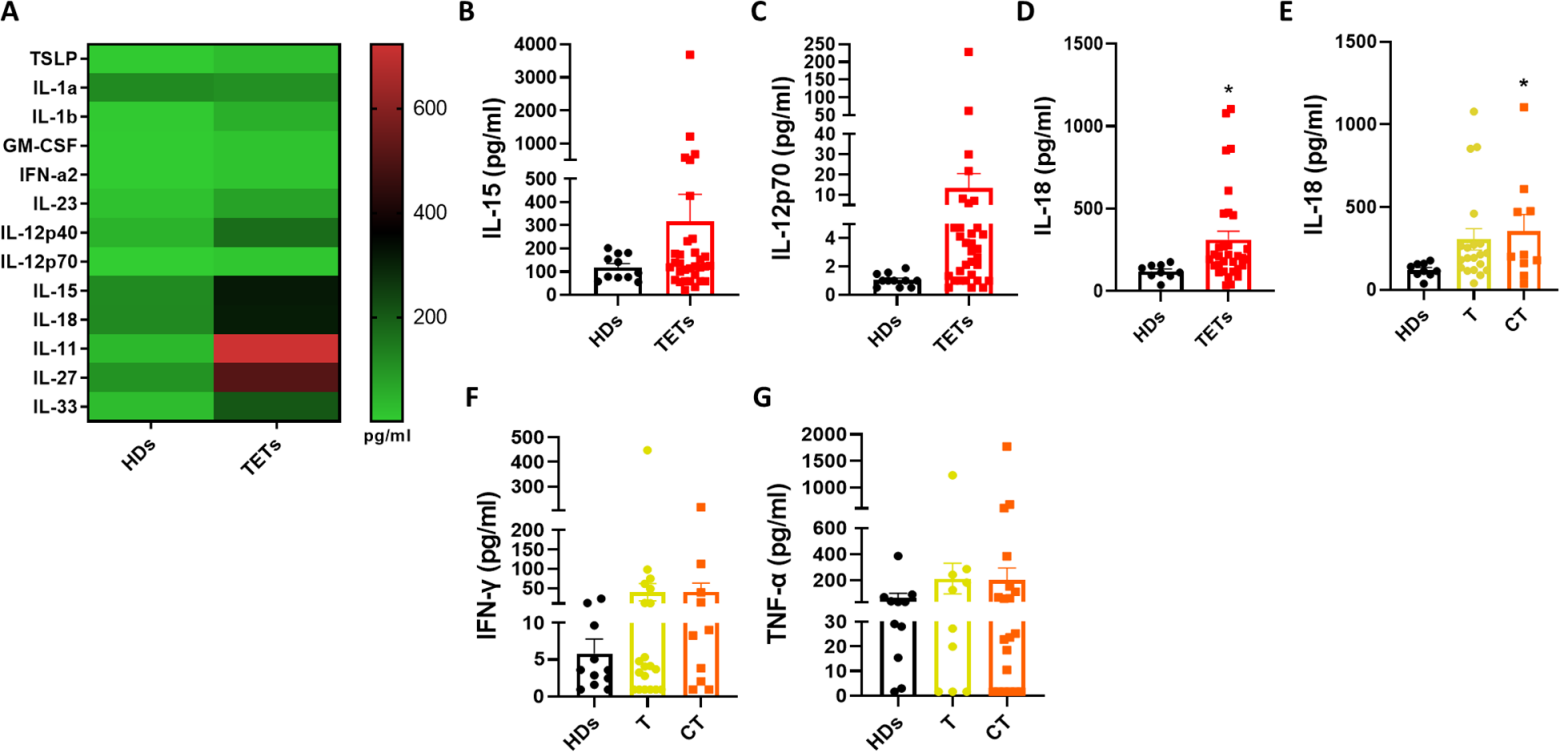
IL-18 is selectively increased in TET patients. **(A)** Quantification of ILC activating cytokines evaluated by multiplex assay in HDs (n= 11) and TETs patients’ sera (n= 32). **(B–D)** Quantification of IL-15, IL-12p70, IL-18 in HDs and TETs patients’ sera. **(E)** Quantification of IL-18 in HDs and TETs patients divided in thymoma (T; n=20) and thymic carcinoma (CT; n=10). **(F, G)** Quantification of IFN-γ and TNF-α assessed by Legendplex™ analysis in HDs and TETs patients’ sera. Data are shown as mean ± SEM (*p <0.05) and were analyzed by Wilcoxon and/or one-way ANOVA tests.

To investigate potential mechanisms underlying ILC1 dysregulation in TETs, we measured serum levels of cytokines known to activate ILCs using a bead-based multiplex immunoassay. A global comparison between healthy donors and TET patients is shown in the heatmap in **Figure 3A**. We then focused on cytokines involved in ILC1 activation, including IL-12p70, IL-15, and IL-18 (**Figures 3B–D**). Among these cytokines, only IL-18 was significantly elevated in the serum of TET patients compared to healthy donors (**Figure 3D**), in line with the observed increase in circulating ILC1 frequency. To further assess this finding, patients were stratified by tumor histology. IL-18 levels were significantly higher in patients with thymic carcinoma, while thymoma patients displayed lower levels (**Figure 3E**). In contrast, the levels of typical ILC1 effector cytokines, such as IFN-γ and TNF-α, did not differ significantly between the two histological subgroups (**Figures 3F, G**). These data suggest that IL-18 upregulation may contribute to ILC1 expansion in TETs, particularly in thymic carcinoma, although it is not accompanied by increased production of canonical ILC1-derived cytokines. Elevated serum IL-18 could represent a quantifiable biomarker of innate immune activation in TETs, especially in more aggressive histological subtypes. To further explore potential relationships among circulating ILC subsets, cytokine levels, and clinical parameters, we performed a Spearman’s correlation analysis (**Supplementary Figure 4**). The correlation matrix highlighted a positive association between ILC1 and IL-18, consistent with the predominance of type 1 inflammatory cues in TET patients. Conversely, ILCP showed an inverse trend with IL-12p40, whereas no positive correlation was observed between ILC2 and type 2 cytokines (IL-4, IL-13), possibly reflecting the overall contraction of this subset in the peripheral blood of TET patients. No consistent correlations emerged between cytokine levels and clinical variables such as histology (thymoma vs thymic carcinoma), sex, or autoimmunity.

### ILC2-associated cytokines are enriched in thymic carcinoma patients

**Figure 4:**
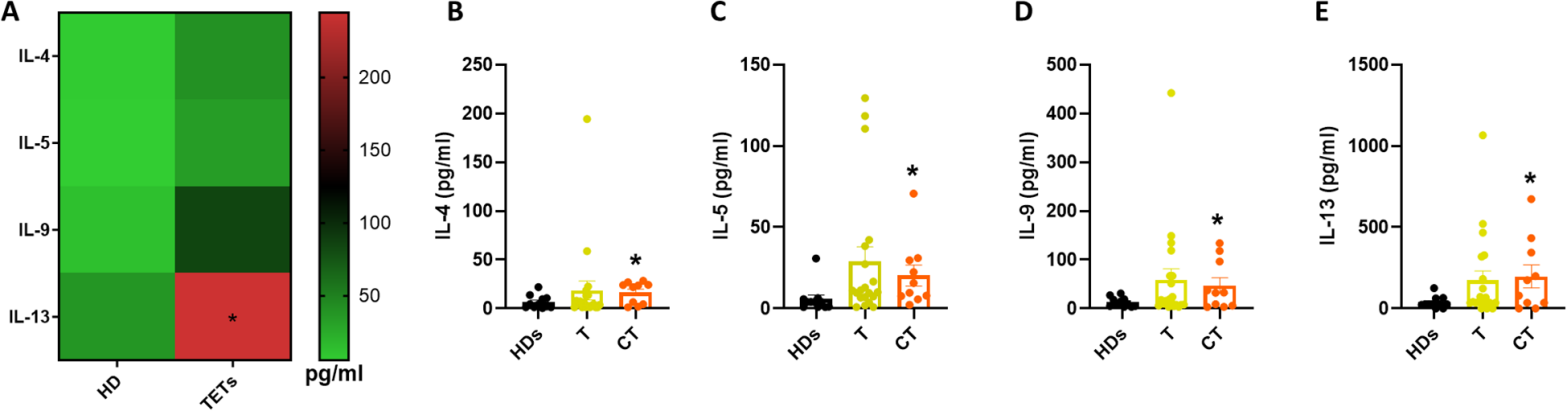
ILC2-associated cytokines are enriched in thymic carcinoma patients. **(A)** Quantification of type 2 cytokines in HDs (n= 11) and TETs patients’ sera (n= 32). **(B–E)** Quantification of IL-4, IL-5, IL-9 and IL-13 in HDs and TETs patients’ sera. Data are shown as mean ± SEM (*p <0.05) and were analyzed by Wilcoxon and/or one-way ANOVA tests.

Given that IL-18 has also been implicated in promoting ILC2 activation under certain inflammatory contexts (15), we next assessed systemic levels of type 2 cytokines typically associated with ILC2 function. Serum concentrations of IL-4, IL-5, IL-9, and IL-13 were measured in both HDs and TET patients (**Figure 4A**). Among the four cytokines analyzed, only IL-13 was significantly elevated in TET patients compared to HDs, while IL-4, IL-5, and IL-9 displayed a non-significant upward trend. To further examine potential associations with tumor histology, patients were stratified into thymoma (T) and thymic carcinoma (CT) groups. This analysis revealed a significant increase in all measured type 2 cytokines in patients with TC compared to HDs (**Figures 4B–E**). These findings suggest a systemic shift toward a type 2 cytokine profile in thymic carcinoma, potentially reflecting increased ILC2 activation in this tumor subtype. The type 2 cytokine enrichment observed in thymic carcinoma may indicate an immune escape pathway that could be exploited for therapeutic targeting.

## Discussion

This study reveals a previously uncharacterized systemic dysregulation of ILCs in thymic tumors, suggesting that these rare neoplasms profoundly alter both the composition and activation state of the innate immune compartment. These findings have direct clinical implications, as IL-18 levels and ILC subset profiling could complement imaging-based follow-up in TETs. While ILCs have been increasingly recognized as modulators of tissue inflammation and cancer immunity (16–18), their role in thymic malignancies has remained unexplored. Our findings indicate a preferential expansion of group 1 ILCs in the peripheral blood of patients, particularly those with evidence of disease and thymic carcinoma, pointing to a potential link between tumor aggressiveness and this innate compartment. This association suggests that ILC1 enumeration could serve as a dynamic marker to monitor tumor activity and aggressiveness in clinical practice. Notably, within the healthy donor group, ILC1 frequencies displayed a certain degree of dispersion, with some individuals showing values close to those observed in TET patients. This observation is in line with previous studies reporting high inter-individual variability in the abundance of circulating ILC subsets in healthy adults (19, 20).

The selective enrichment of ILC1s in the absence of a parallel increase in canonical effector cytokines such as IFN-γ and TNF-α suggests a state of functional decoupling as observed in other cancer types including melanoma and lung cancer (18, 21, 22). Among the serum cytokines assessed, IL-18 emerged as the most significantly elevated and may represent a key driver of ILC1 expansion (23). However, the absence of a parallel increase in ILC1 effector cytokines despite elevated IL-18 levels suggests the involvement of inhibitory pathways, such as IL-18BP—which directly neutralizes IL-18—and IL-37, which forms a complex with IL-18BP and IL-18Rα, thereby enhancing IL-18BP–mediated suppression and inhibiting IL-18–driven responses (24, 25). Targeting the IL-18/IL-18BP/IL-37 axis could represent a novel immunotherapeutic approach in aggressive disease. Nevertheless, whether these tumors promote such decoupling directly or whether it reflects a broader immunoregulatory program induced by the microenvironment remains to be clarified. Unexpectedly, thymic carcinoma cases exhibited a distinct cytokine milieu skewed toward type 2 inflammation, with increased levels of IL-4, IL-5, IL-9, and IL-13. Although overall ILC2 frequencies were not significantly changed, this profile may reflect a functional polarization of ILC2s. Importantly, IL-18 has also been implicated in activating ILC2 responses in chronic myeloid leukemia and prostate cancer (9, 15) promoting immune suppression and tumor progression, raising the possibility that similar mechanisms may be involved also in thymic carcinoma. This systemic shift toward type 2 inflammation could be integrated into patient stratification and may influence responsiveness to immune checkpoint blockade or cytokine-targeted therapies. In addition, modest increases of IL-11 and IL-27 were detected, although they did not reach statistical significance. Notably, IL-27 has been described as an immunoregulatory cytokine capable of restraining excessive ILC activation, particularly within type-2 responses (26, 27). In our context, the mild elevation of IL-27 might reflect a compensatory mechanism aimed at counterbalancing the enhanced type-2 cytokine milieu observed in TET patients. The thymic microenvironment may contribute to ILC differentiation and activation. Differences between tumor subtypes, although not statistically significant, warrant investigation in larger cohorts. In addition, some patients were under systemic treatment or corticosteroid therapy at the time of blood collection, which may have influenced immune readouts. Expanding the cohort size in future studies will be crucial not only to validate these findings, but also to better dissect how treatment-related and thymic factors collectively shape ILC homeostasis. Nevertheless, our findings delineate a distinct pattern of innate immune dysregulation in TETs, marked by subset-specific alterations and cytokine profiles that are particularly enriched in aggressive disease. Given the accessibility of peripheral blood analysis, prospective integration of IL−18 quantification and ILC profiling into TET management could refine risk assessment, guide therapeutic decisions, and monitor recurrence.

## Data Availability

The raw data supporting the conclusions of this article will be made available by the authors, without undue reservation.
