# Supplementary material for: Circulating innate lymphoid cells and IL-18 as potential immune biomarkers in thymic tumors
Source: Front Immunol. 2025 Nov 24;16:1705648. doi: 10.3389/fimmu.2025.1705648 (PMC12682857; doi:10.3389/fimmu.2025.1705648)
Supplement: Supplementary file 1 [file SupplementaryFile1.docx]

**Supplementary Figures**

**
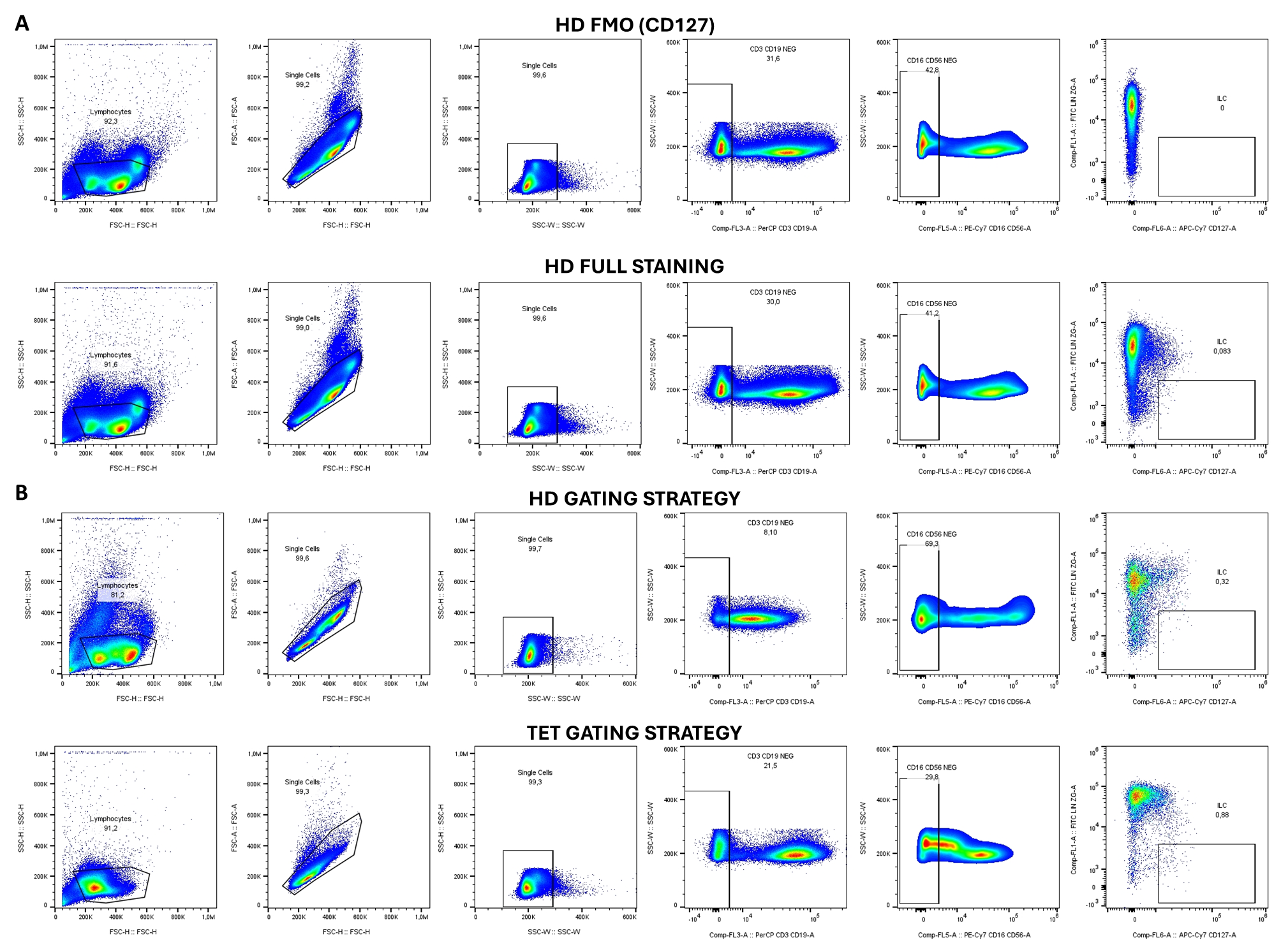
**

**Supp. Figure 1. (A)** Representative CD127 FMO and full staining in a healthy donor (HD) used to define the CD127⁺ threshold. **(B)** Complete gating strategy for ILC identification in representative HD and TET samples. The workflow includes: lymphocyte selection (FSC/SSC), doublet exclusion, gating on CD3⁻ CD19⁻ and CD16⁻ CD56⁻ cells to exclude T-cell and NK-cell contamination, and identification of ILCs as Lineage⁻ Zombie Green⁻ CD127⁺ cells. The Zombie Green viability dye, included together with lineage markers in the FITC channel, allowed joint exclusion of dead and Lineage⁺ cells in the FITC⁺ fraction without affecting the detection of live ILCs. The same gating strategy and CD127 threshold (defined from the FMO) were applied to all samples in both groups.


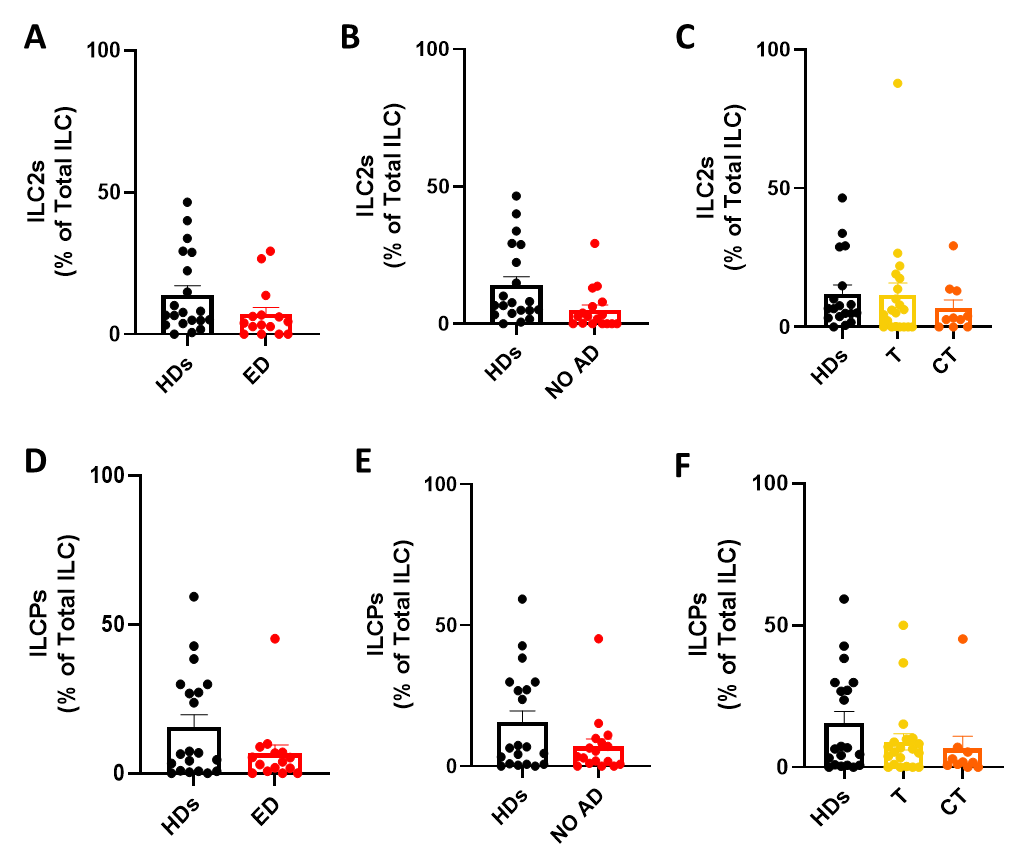


******

**Supp. Figure 2. (A)** Frequency of ILC2s in HDs and patients with evidence of disease (ED; n=15). **(B)** Frequency of ILC2s in HDs and patients without autoimmune disorders (NO AD; n=17). **(C)** Frequency of ILC2s in TETs patients divided in thymoma (T; n=20) and thymic carcinoma (CT; n=10). **(D)** Frequency of ILCPs in HDs and patients with evidence of disease (ED; n=15). **(E)** Frequency of ILCPs in HDs and patients without autoimmune disorders (NO AD; n=17). **(F)** Frequency of ILCPs in TETs patients divided in thymoma (T; n=20) and thymic carcinoma (CT; n=10). Data are shown as mean ± SEM (** p < 0.01 vs HDs) and were analysed by Wilcoxon and/or one-way ANOVA tests.


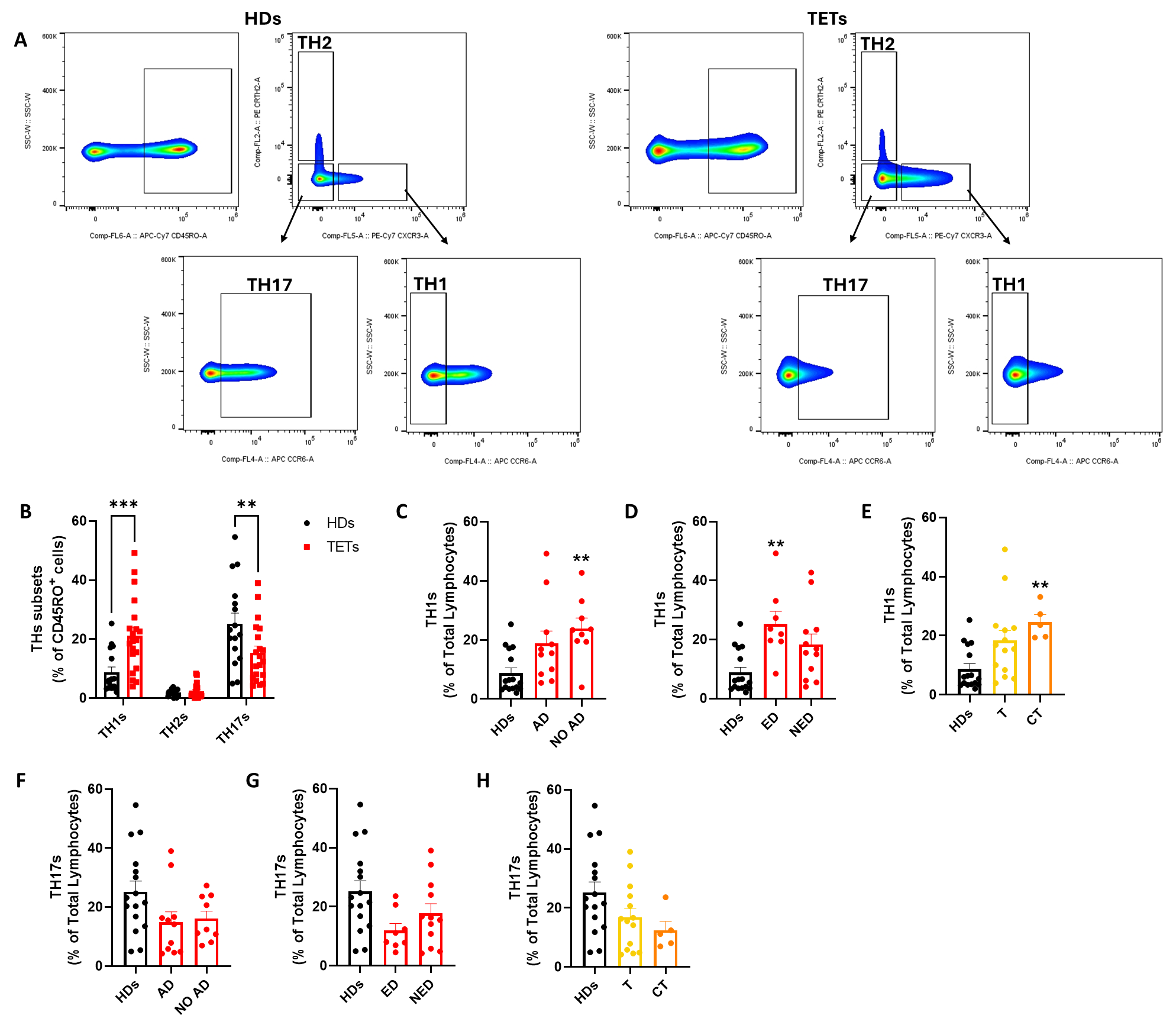


**Supp. Figure 3. (A)** Representative examples of flow cytometry analysis of Th subsets in PBMCs of HDs (left panel) and TETs patients (right panel). (**B**) Frequency of Th subsets in HD and TETs patients. **(C)** Frequency of Th1s in HDs and patients with or without autoimmune disorders. **(D)** Frequency of Th1s in HDs and patients with or without evidence of disease. **(E)** Frequency of Th1s in TETs patients divided in thymoma and thymic carcinoma. **(F)** Frequency of Th17s in HDs and patients with or without autoimmune disorders. **(G)** Frequency of Th17s in HDs and patients with or without evidence of disease. **(H)** Frequency of Th17s in TETs patients divided in thymoma and thymic carcinoma. Data are shown as mean ± SEM (** p < 0.01; *** p < 0.001 vs HDs) and were analysed by Wilcoxon and/or one-way ANOVA tests.

**Supp. Figure 4.** Spearman correlation analysis including circulating ILC1, ILC2, and ILCp frequencies, plasma cytokine levels, and clinical parameters (sex, autoimmunity, disease status, and histology). Blue indicates positive and red negative correlations.
